# Relationship Between Dry Eye Disease and Emotional Disorder: The Mediating Effect of Health Anxiety

**DOI:** 10.3389/fpubh.2022.771554

**Published:** 2022-02-28

**Authors:** Qing He, Zhuo Chen, Caiyuan Xie, Lin Liu, Haibo Yang, Ruihua Wei

**Affiliations:** ^1^Tianjin Key Laboratory of Retinal Functions and Diseases, Tianjin Branch of National Clinical Research Center for Ocular Disease, Eye Institute and School of Optometry, Tianjin Medical University Eye Hospital, Tianjin, China; ^2^Academy of Psychology and Behavior, Tianjin Normal University, Tianjin Social Science Laboratory of Students' Mental Development and Learning, Tianjin, China

**Keywords:** anxiety, depression, dry eye disease, emotion, health anxiety

## Abstract

**Objective:**

This study aims to investigate the relationship between dry eye disease (DED) and anxiety, as well as DED and depression. Additionally, the influence of health anxiety (HA) on this relationship was determined.

**Methods:**

A total of 206 patients with DED were recruited from Tianjin Medical University Eye Hospital clinic and surveyed using demographic questionnaires, the Ocular Surface Disease Index (OSDI), Hospital Anxiety and Depression Scale (HADS), and Short Health Anxiety Inventory (SHAI). Additionally, they were examined using Keratograph 5M and assessed for DED by corneal fluorescein staining. Regression analysis and the bootstrap method were used to investigate the influence of HA on the relationship between DED and emotional disorders.

**Results:**

Among the 206 patients with DED, 52 (25.24%) and 56 (27.18%) patients showed depression and anxiety, respectively. The OSDI score and HA were positively correlated with depression and anxiety (*P* < 0.01). The direct effects of OSDI on depression and anxiety were significant (95% confidence interval [CI]: 0.017–0.069; 0.008–0.060). Additionally, the bootstrap test showed significant mediating effects of HA (95% CI: 0.001–0.016; 0.003–0.021). The results suggested that the severity of DED symptoms, as measured by the OSDI score, affected anxiety and depression by a direct and an indirect pathway mediated by HA.

**Conclusions:**

We found a significant correlation between DED and anxiety and depression. Moreover, HA was a mediator of the relationship between DED symptoms and anxiety and depression.

## Introduction

Dry eye disease (DED) is a common ocular surface disease with a prevalence ranging from 5.3 to 50.1% and 7.99 to 29.9% in community and hospital-based studies, respectively (1–4). In 2017, DED was redefined as a multifactorial disease of the ocular surface characterized by a loss of homeostasis of the tear film accompanied by ocular symptoms, in which tear film instability and hyperosmolarity, ocular surface inflammation and damage, and neurosensory abnormalities play etiological roles (5). In some cases, patients complain of discomfort including dryness, foreign body sensation, burning sensation, photophobia, and eye pain; however, they exhibit less severe ocular signs. In addition, several studies have shown a relationship between symptoms of DED and emotional disorder, but there is no correlation between changes in ocular surface parameters of DED and emotional disorder (6–8). Considering this, DED has gradually become a “biopsychosocial” issue and the concern of physicians regarding the emotional and mental state of patients with DED has increased (6, 9, 10).

Several studies have reported a correlation between DED and mental illness, including depression and anxiety. As compared with other ophthalmic diseases, patients with DED are more likely to experience depression (11). These studies mostly show that the symptoms of DED are associated with anxiety and depression (8, 10, 12). Moreover, discomfort symptoms would impact the quality of life of such patients (13, 14). Currently, the prevention and treatment of emotional disorders in patients with DED are not well-studied. Therefore, the investigation of factors affecting the development of anxiety and depression in patients with DED is necessary to develop effective and comprehensive treatments.

Health anxiety (HA) is a patient's concern regarding their health because of misunderstandings regarding the physical sensations or changes (15). It is conceptualized as a continuum that ranges from mild non-clinical health to severe or hypochondriacal concerns (16–18). The prevalence of HA in general hospitals is approximately 9% and usually affects middle-aged and older adults (18). HA is closely associated with chronic diseases including asthma, rheumatoid arthritis, and diabetes (19, 20). Several studies have reported a correlation between HA and the emotional state (18, 21). However, few studies have explored the influence of HA on the relationship between DED, anxiety, and depression.

The aim of this study was to investigate the anxiety and depression status of clinical patients with DED and to explore the mediating role of HA in the relationship between DED, anxiety, and depression.

## Materials and Methods

In total, 206 patients with DED were recruited from May 2021 to July 2021 at Tianjin Medical University Eye Hospital. Approval was obtained from the Medical Ethics Committee of Tianjin Medical University Eye Hospital. The procedures used in this study adhere to the tenets of the Declaration of Helsinki. This study included literate adults aged >18 years with an OSDI score ≥13 points (22). We excluded patients who had complications from anterior ocular segment diseases except for DED. Patients with autoimmune diseases, severe cardiopulmonary diseases, allergic disease and neurologic or psychiatric disorders, including those previously diagnosed with anxiety and depression, were excluded. In addition, patients who received anti-allergy drugs and contraceptives were excluded. Patients with an alcohol and drug dependence history, those with anti-anxiety and antidepressant drugs, those with serious medical conditions that prevented them from participating in the questionnaire, those who were unable to care for themselves, those who were severely illiterate, and pregnant and lactating women were also excluded. Informed consent was obtained from all individual participants included in the study. Demographic and medical data were collected.

### Evaluation of DED

First, all participants accomplished the Ocular Surface Disease Index (OSDI) questionnaire, a self-administered questionnaire that assesses the severity of self-reported DED. Based on the total OSDI score, each participant's condition was classified as normal (0–12 points), mild (13–22 points), moderate (23–32 points), and severe (33–100 points). A score of ≥13 points led to a diagnosis of DED (22).

The participants were tested using the Keratograph 5M (Oculus, Wetzlar, Germany) to evaluate the objective indicators of DED (23). The tests were performed by a skilled physician at our hospital. A standardized room was used for the examination of patients. The patient was asked to place the lower jaw on the analyzer bracket. The height of the elevator and the bracket was adjusted according to the patient's condition until the patient's eyes were close to the Placido ring. Each patient was instructed to look at the red light in the center of the ring in front of him/her, blink three times, and then open the eyes as wide as possible. At this point, the physician performed a quick examination, capturing and analyzing images automatically. The upper and lower eyelids were everted, and more images were captured and analyzed.

Measurements included the non-invasive mean tear breakup time, non-invasive first tear breakup time (NIBUT), tear meniscus height, and meibomian glands loss rate. Partial or complete loss of the meibomian glands was recorded as grade 0 (no loss of meibomian glands), 1 (gland dropout was <1/3 of the total meibomian glands), 2 (gland dropout was 1/3–2/3 of the total meibomian glands), and 3 (gland dropout was >2/3 of the total meibomian glands) for each eyelid (24). Finally, the same physician performed the slit-lamp examination of the anterior and posterior ocular segments in order to exclude some ocular diseases mentioned above.

The corneal staining scores were measured using commercially available pre-packaged sterile fluorescein paper tape (Jingming New Technology Development Co. Ltd., Tianjin, China). Corneal staining was scored according to the following criteria (25): The corneas were divided into the upper, middle, and lower regions. No staining = 0 points; <5 staining spots = 1 point; 5–9 staining spots = 2 points; ≥10 staining spots staining or filiform staining = 3 points. The total corneal staining score was calculated as the sum of the scores of all three corneal regions, ranging from 0 to 9.

### HA Assessment

The Short Health Anxiety Inventory (SHAI) questionnaire was developed by Salkovskis et al. and is widely used to measure the HA levels (26), with a validity and reliability of 0.76 and 0.89, respectively. Each item consists of four declarative statements representing different levels of illness, scored from 0 to 3 as follows: 0, low level; 1, mild level; 2, moderate level; and 3, severe level. The total score ranges from 0 to 54, with higher scores representing higher levels of HA. The SHAI has good reliability and validity values and is widely used in screening for HA (27). In this study, the reliability was 0.61.

### Emotion Status Assessment

The Hospital Anxiety and Depression Scale (HADS) questionnaire was created by Zigmond and Snaith (28). It mainly screens anxiety and depression among patients in general hospitals. The scale contains 14 items, divided into two subscales containing seven items each to assess depression and anxiety. The scale is scored on a 4-point scale (0–3), and the cut-off points for anxiety and depression are total scores of 8 points. HADS has good reliability and validity values (29), and is widely used in screening for anxiety and depression. In this study, the reliability was 0.87.

### Statistical Analysis

Statistical analysis was performed using the SPSS software (version 23.0; IBM Corp., Armonk, NY, USA). The χ^2^-test was used to compare the demographic data. When the theoretical frequency was 1 ≤ T <5, a corrected χ^2^-test was used. As the data were normally distributed, we used Pearson correlation to analyze the correlations between the OSDI questionnaire, ophthalmic data, HA, and HADS scores. Multiple linear hierarchical regression models were applied to test the mediating role of HA in the relationship of DED with anxiety, and depression, using household location and duration of illness as control variables. Prior to the mediation analysis, all continuous variables were pooled to eliminate multicollinearity. The mediating role of HA was further tested using the SPSS bootstrap analysis of 5,000 samples. All tests were two-sided, and statistical significance was set at *P* < 0.05.

## Results

This study included 206 patients with DED (47 male and 159 female patients). The general information of the patients is presented in Table 1. Among them, 52 (25.24%), 56 (27.18%), and 39 (18.93%) patients presented with depression, anxiety, and both depression and anxiety, respectively.

**Table 1:** Comparison of depressive symptoms and anxiety symptoms between patients with dry eye.

**Group**	**Depression (*n* = 52)**	**Normal (*n* = 154)**	**χ^2^**	***P***	**Anxiety (*n* = 56)**	**Normal (*n* = 150)**	**χ^2^**	***P***
**Sex**			0.003	0.959			1.986	0.159
Male	12 (25.53%)	35 (74.47%)			9 (19.15%)	38 (80.85%)		
Female	40 (25.16%)	119 (74.84%)			47 (29.56%)	112 (70.44%)		
**Menstruation**			1.307	0.253			0.809	0.368
Menopause	25 (28.74%)	62 (71.26%)			28 (32.56%)	58 (67.44%)		
Non-menopause	15 (20.83%)	57 (79.17%)			19 (26.02%)	54 (73.98%)		
**Marital status**			2.225	0.136			2.959	0.085
Married	48 (27.43%)	127 (72.57%)			52 (29.71%)	123 (70.29%)		
Single	4 (12.90%)	27 (87.10%)			4 (12.90%)	27 (87.10%)		
**Household location**			7.021	0.009			3.091	0.049
Urban	36 (21.43%)	132 (78.57%)			41 (24.40%)	127 (75.60%)		
Countryside	16 (42.11%)	22 (57.89%)			15 (39.47%)	23 (60.53%)		
**BMI**			2.078	0.556			1.961	0.580
<18.5	1 (9.09%)	10 (90.91%)			1 (9.09%)	10 (90.91%)		
18.5 ≤ BMI <25	34 (27.64%)	89 (72.36%)			35 (28.46%)	88 (71.54%)		
25 ≤ BMI <30	15 (24.19%)	47 (75.81%)			17 (27.42%)	45 (72.58%)		
≥30	2 (20.00%)	8 (80.00%)			3 (30.00%)	7 (70.00%)		
**Course of disease**			11.442	0.010			6.864	0.076
≤ 1 year	14 (15.56%)	76 (84.44%)			18 (20.00%)	72 (80.00%)		
1–3 year	24 (29.63%)	57 (70.37%)			23 (28.40%)	58 (71.60%)		
3–5 year	7 (31.82%)	15 (68.18%)			9 (40.90%)	13 (59.10%)		
≥5 year	7 (53.85%)	6 (46.15%)			6 (46.15%)	7 (53.85%)		
**Frequency of visit (within 1 year)**			5.502	0.139			4.947	0.176
First visit	18 (22.50%)	62 (77.50%)			20 (25.00%)	60 (75.00%)		
<6 times	20 (21.74%)	72 (78.26%)			22 (23.91%)	70 (53.85%)		
6–12 times	10 (41.67%)	14 (58.33%)			11 (45.83%)	13 (54.17%)		
<12 times	4 (40.00%)	6 (60.00%)			3 (30.00%)	7 (70.00%)		

We found that rural residents were more likely to have anxiety and depression compared to urban residents (*P* < 0.05). Furthermore, the duration of the disease was another factor that was associated with depression (*P* < 0.05).

The mean OSDI score was 47.69 ± 17.01 points. We found that 146 (75.73%) patients with DED were categorized as severe according to the OSDI scoring. In total, 17 (8.24%) and 33 (16.02%) patients were categorized as mild and moderate, respectively.

The results of the correlation analyses are presented in Table 2. The OSDI score was significantly correlated with anxiety, depression, and HA. Anxiety was correlated with depression and HA. Depression was correlated with HA. Meibomian glands loss was correlated with depression and HA. No significant correlation was observed among the tear meniscus height, NIBUT, and corneal staining scores.

**Table 2:** Correlation between depression, anxiety, SHAI, OSDI, and ophthalmologic parameters.

	**OSDI**	**TMH**	**NITBUT**	**MGL**	**CSS**	**Anxiety**	**Depression**	**SHAI**
OSDI	-							
TMH	−0.084	-						
NIBUT	−0.136	0.034	-					
MGL	0.169^*^	−0.032	−0.096	-				
CSS	0.337^**^	−0.108	−0.215^**^	−0.096	-			
Anxiety	0.236^**^	0.048	0.081	0.133	0.082	-		
Depression	0.253^**^	−0.098	0.015	0.181^*^	0.089	0.716^**^	-	
SHAI	0.218^**^	−0.050	0.006	0.139^*^	−0.057	0.288^**^	0.277^**^	-

* *P < 0.05,*

** *P < 0.01. SHAI, Short Health Anxiety Inventory; OSDI, Ocular Surface Disease Index; TMH, Tear Meniscus Height; NIBUT, non-invasive first tear breakup time; MGL, Meibomian Glands loss; CSS, Corneal Staining Scores*.

**Figure 1:**
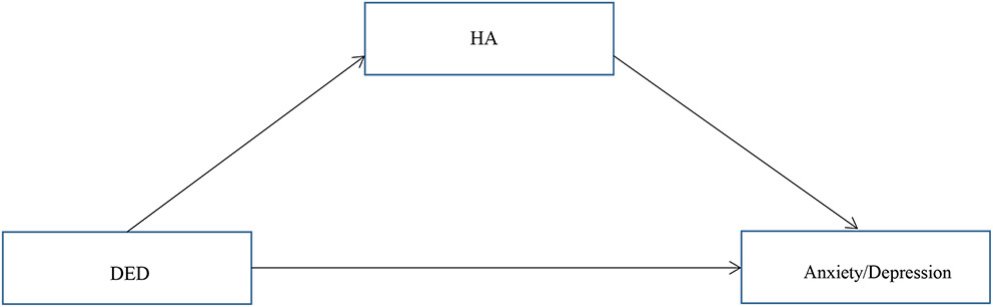
The proposed model of relationships between variables.

Our primary hypothesis (Figure 1) was that the association of DED severity with depressive and anxiety symptoms would be mediated by HA. OSDI scores and HA were positively correlated with depression and anxiety (*P* < 0.01), and the conditions for conducting a mediation analysis were satisfied. Hierarchical regression analysis (Table 3) revealed that the OSDI score was positively correlated with HA, depression, and anxiety (*P* < 0.05). HA was positively correlated with depression and anxiety (*P* < 0.05). The direct effects of the severity of DED, as measured by the OSDI scores, on depression and anxiety were significant (95% confidence interval [CI]: 0.017–0.069; 0.008–0.060). Additionally, the bootstrap test (Table 4) showed that the mediating effects of HA were significant (95% CI: 0.001–0.016; 0.003–0.021).

**Table 3:** Mediation analysis of SHAI on the relationship between OSDI, depression, and anxiety.

	***B***	**SE**	***t***	***P***
OSDI on depression	0.052	0.014	3.734	0.000
OSDI on SHAI	0.057	0.018	3.186	0.002
SHAI on depression (indirect effect)	0.181	0.053	3.449	0.001
OSDI on depression (direct effect)	0.041	0.014	2.990	0.003
OSDI on anxiety	0.046	0.013	3.472	0.001
OSDI on SHAI	0.057	0.018	3.186	0.002
SHAI on anxiety (indirect effect)	0.183	0.050	3.675	0.000
OSDI on anxiety (direct effect)	0.035	0.013	2.691	0.008

*SHAI, Short Health Anxiety Inventory; OSDI, Ocular Surface Disease Index*.

**Table 4:** Bootstrap results for the mediation analysis.

**Variables**	**Estimate**	**SE**	**LL95%CL**	**UL95%CL**
OSDI on depression (direct effect)	0.045	0.210	0.017	0.069
SHAI on depression (indirect effect)	0.006	0.029	0.001	0.016
OSDI on anxiety (direct effect)	0.035	0.013	0.008	0.060
SHAI on anxiety (indirect effect)	0.011	0.004	0.003	0.021

*SHAI, Short Health Anxiety Inventory; OSDI, Ocular Surface Disease Index*.

## Discussion

This study evaluated the prevalence of depression and anxiety among patients with DED and investigated the factors affecting this relationship. Approximately 25.24 and 27.18% of patients with DED presented with anxiety and depression, respectively. Previous studies in different populations have showed consistent results. Zheng et al. (11) found that the prevalence of depression in patients with DED (27%) was significantly higher than that of other ocular diseases. Another study from Turkey found that the prevalence of depression and anxiety in patients with DED was 53.7 and 63.6%, respectively (30). In outpatient clinics, Wu et al. (6) found 46% prevalence of depression and 39% prevalence of anxiety in patients with DED. In DED clinics, depression was observed in 61% of patients (9). The difference in our findings may be attributed to the difference in depression and anxiety measurement methods and inconsistencies in the population involved. In addition, we found that the course of disease in patients with DED was a factor influencing depression. However, the trend between them was unclear because of the small number of depressed patients with a long disease course in our results.

In our study, we found a significant correlation between the OSDI scores, anxiety, and depression in patients with DED; however, no correlation was observed between the tear meniscus height, NIBUT, and corneal staining score. Similarly, Kim et al. (8) found a significant correlation between the subjective symptoms of DED and depression but not with the results of other ophthalmologic examinations. However, several studies have not found a significant correlation between the OSDI score and depression (9, 31, 32). We believe that the inclusion of fewer patients with DED in these works might have led to this observed difference; moreover, these studies also used different questionnaires to evaluate anxiety and depression. The causal relationship between DED and depression is unclear. However, some factors have been used to explain their association. First, the two diseases are homologous (33); especially, both share common risk factors, including female sex and menopause. This suggested that sex hormones play an important role in the development of both diseases. Second, somatization, a common symptom of depression, may play a role in exacerbating DED symptoms (34). Somatization is present in 80% of patients with depression (35). The strong association of depression with DED symptoms, but not DED signs, support this notion.

Studies have shown a separation between the symptoms and signs of DED (36, 37). Olaniyan et al. (38) used the OSDI to evaluate DED, according to which the prevalence of DED was significantly higher than that observed with objective examination. This finding was consistent with that observed in our study. Subjective symptom scores of DED patients are positively correlated with depression and anxiety. Therefore, the emotional state of these patients should be assessed in addition to ophthalmological examination. In this study, the mediation effect of HA on the relationship of DED, anxiety, and depression was evaluated.

People with HA are more sensitive to physical symptoms. This may be attributed to the negative events they encounter, causing them to exhibit stress reactions. Therefore, these individuals tend to visit the hospital more frequently than others (39). In this study, we found that the severity of DED symptoms, as measured by the OSDI score, affected anxiety and depression by a direct and an indirect pathway mediated by HA. Researchers have found that some factors, including personality, emotional regulation, and cognitive factors, play an important role in the development of HA (21, 40). People with high HA are more sensitive to present somatic symptoms than normal, and they are more likely to develop concerns and fears concerning their somatic performance that may arise from their irrational perceptions and beliefs. Moreover, they are often unable to distinguish their emotional experiences from somatic feelings, thus leading to excessive concerns regarding the somatic sensations and repeated seeking of medical help (41). People with high levels of HA, after experiencing eye discomfort for a period, consider DED as an incurable disease and gradually shift to a negative coping style, during which they often do not follow medical advices and medication regimens, leading to worsening conditions and low recovery expectations (42). HA can be interpreted as an individual's susceptibility to and misunderstanding of physical symptoms (26, 27). The identification and management of underlying psychological disorders may complement clinical treatment to improve patients' subjective symptoms and enhance their quality of life.

This study had several limitations. First, this was a cross-sectional study, and a causal relationship among DED, anxiety, depression, and HA was not established. In the future, we could demonstrate whether there is a causal relationship between them through cohort studies. Second, this study was subjective because it relied on a self-assessment questionnaire. In addition, the HADS questionnaire is a screening tool for anxiety and depression. Thus, patients suspected of having anxiety and depression should be further examined to make a definite diagnosis and administer corresponding treatment. The scale should not be used as a diagnostic tool in epidemiological investigation research (43). Third, the patients were from a single region (Tianjin, China). Undoubtedly, sociocultural factors play an important role in the development of the disease; therefore, the generalizability of this study might have been affected. A prospective study involving multiple locations and centers and a larger population is needed to confirm and expand the findings. Fourth, future studies should include psychological mediating variables, such as personality, in addition to HA, for a more comprehensive and in-depth exploration.

## Conclusions

This study showed that many patients with DED experience anxiety and depression. HA was a mediator of the relationship between DED symptoms and anxiety and depression. Therefore, behavioral interventions to develop a correct perception of DED and reduce the level of HA are necessary. These interventions may reduce the anxiety and depression of patients with DED and improve the effectiveness of DED treatment.

## Data Availability Statement

The raw data supporting the conclusions of this article will be made available by the authors, without undue reservation.

## Ethics Statement

The studies involving human participants were reviewed and approved by Tianjin Medical University Eye Hospital No: 2020KY(L)-17. The patients/participants provided their written informed consent to participate in this study.

## Author Contributions

Material preparation, data collection and analysis were performed by QH, ZC, and CX. The first draft of the manuscript was written by QH. All authors contributed to the study conception and design and commented on previous versions of the manuscript. All authors read and approved the final manuscript.

## Funding

This work was supported by a grant from the National Natural Science Foundation of China (Grant no: 81770901).

## Conflict of Interest

The authors declare that the research was conducted in the absence of any commercial or financial relationships that could be construed as a potential conflict of interest.

## Publisher's Note

All claims expressed in this article are solely those of the authors and do not necessarily represent those of their affiliated organizations, or those of the publisher, the editors and the reviewers. Any product that may be evaluated in this article, or claim that may be made by its manufacturer, is not guaranteed or endorsed by the publisher.
